# Inorganic polyphosphate chelates Ca^2+^ but cannot substitute for ATP as an energy source for SERCA

**DOI:** 10.1016/j.bbrep.2026.102796

**Published:** 2026-09-14

**Authors:** F.A. Khasanov, F.R. Rustamova, E.P. Semernya, L.M. Akhmadkhojaeva, K.S. Nebesnaya, K.R. Rustamov, A.Y. Baev

**Affiliations:** a Center for Advanced Technologies, Academy of Sciences of the Republic of Uzbekistan, Tashkent, Uzbekistan; b Department of Pharmacy and chemistry, Faculty of Dentistry and Pharmacy, Alfraganus University, Uzbekistan

**Keywords:** Inorganic polyphosphate, SERCA, Sarcoplasmic reticulum, ADP, Ca^2+^ chelation, Thapsigargin

## Abstract

Inorganic polyphosphate (polyP) is a high-energy polymer with phosphoanhydride bonds analogous to ATP, but whether it can serve as a phosphoryl donor for mammalian ATP-dependent enzymes remains unclear. Because PMCA was previously reported to utilize polyP in human erythrocytes, we tested whether the closely related sarco/endoplasmic reticulum Ca^2+^-ATPase (SERCA) can use polyP to drive Ca^2+^ transport or support polyphosphate kinase-like activity. Protein BLAST identified SERCA as the closest non-PMCA homolog in the human proteome, and docking simulations predicted polyP-14 binding to SERCA with affinities comparable to ATP. However, functional assays with isolated SR vesicles showed that polyP neither supported SERCA-mediated Ca^2+^ uptake nor detectable polyphosphate kinase-like activity. Instead, fluorescence assays and 500 ns molecular dynamics simulations demonstrated direct Ca^2+^ chelation by polyP, with stable coordination of 7–9 Ca^2+^ ions per polyP-14 chain. Unexpectedly, ADP, but not AMP, induced concentration-dependent, thapsigargin-sensitive Ca^2+^ uptake. Thus, polyP is a potent Ca^2+^ chelator but cannot substitute for ATP as an energy source for SERCA.

## Highlights

• PolyP binds SERCA *in silico* but does not support Ca^2+^ transport.
• SERCA shows no detectable polyphosphate kinase-like activity.
• PolyP lowers free Ca^2+^ through direct chelation, independently of SERCA.
• ADP, but not AMP, induces thapsigargin-sensitive Ca^2+^ uptake.

## 1 Introduction

Inorganic polyphosphate (polyP) is a ubiquitous cellular metabolite whose intracellular levels vary under physiological and pathological conditions. Intracellular polyP has been implicated in several processes, including protein polyphosphorylation [1] and chaperone-like functions [[2], [3], [4], [5]]. Upon release into the extracellular space, polyP can act as a signaling molecule involved in blood coagulation [6,7], inflammation [8], and intercellular communication through activation of P2Y1 [[9], [10], [11], [12], [13]], P2X [14], and RAGE receptors [15].

PolyP is a linear polymer of orthophosphate residues linked by phosphoanhydride bonds that are structurally analogous to those of ATP. In mammalian mitochondria, polyP synthesis is driven by the proton-motive force across the inner mitochondrial membrane and depends on active F_O_F_1_-ATP synthase, the same molecular machinery responsible for ATP production. This suggests that polyP and ATP may share a common energetic source and represent alternative products of mitochondrial oxidative phosphorylation [[16], [17], [18], [19]]. Consistent with this concept, depletion of mitochondrial polyP by mitochondria-targeted yeast exopolyphosphatase (MitoPPX) induces a metabolic shift towards glycolysis, increases the ADP/ATP ratio, and alters AMP-activated protein kinase (AMPK) signaling through mechanisms associated with changes in inorganic phosphate (Pi) homeostasis [[20], [21], [22], [23]].

In bacteria and yeast, polyphosphate kinases, including PPK1 and PPK2, catalyze the reversible transfer of terminal phosphate groups from polyP to ADP, thereby regenerating ATP. However, no definitive mammalian orthologue of polyphosphate kinase has been identified to date [24,25]. Therefore, it remains unclear whether polyP can directly serve as a phosphate donor for ADP phosphorylation in mammalian cells.

Several mammalian enzymes have nevertheless been proposed to use polyP as a phosphoryl donor. The plasma membrane Ca^2+^-ATPase (PMCA) in human erythrocytes [26] and NAD kinase [27,28] have been reported to utilize polyP in direct phosphotransfer reactions. In addition, the combined activities of alkaline phosphatase (ALP) and adenylate kinase (AK) can support extracellular ATP and ADP synthesis from polyP [29,30]. PolyP hydrolysis in mammalian cells is mediated by several enzymatic systems, including mitochondrial F_O_F_1_-ATP synthase [18,24] and the Zn^2+^-dependent endopolyphosphatase Nudt3 [31].

Collectively, these observations indicate that polyP may act not only as a signaling molecule and metabolic regulator, but also as a potential source of transferable phosphate energy. However, whether this energy can be directly utilized by mammalian ATP-dependent transport systems remains unresolved.

To address this question, in the current research we investigated whether sarco/endoplasmic reticulum Ca^2+^-ATPase (SERCA) can use polyP as a direct energy source to support Ca^2+^ transport and whether SERCA can exhibit polyphosphate kinase-like activity, as previously reported for PMCA.

## 2 Materials and methods

### 2.1 In silico methods

ATPases similar to PMCA (UniProt: P20020) were identified using protein BLAST search restricted to the human proteome (ranked according to their E-values).

The full-length structures of human SERCA1-3 were predicted with AlphaFold2 [32]. As masked templates, six rabbit SERCA crystal structures representing major catalytic states were employed: E1 (3W5A), E1–2Ca2^+^–ATP (3AR2), E1P–2Ca^2+^–ADP (3BA6), E2P (3B9B), E2–Pi (1WPG), and E2 (3W5C) [[33], [34], [35]].

Molecular docking and molecular dynamics (MD) simulations were performed using AutoDock Vina and GROMACS as described in Refs. [13,14]. Full methodological procedures can be found in supplementary files.

### 2.2 Determination of SERCA activity

Microsomal vesicles from rabbit hindlimb tight muscle were prepared using methods described in Ref. [36].

SERCA activity was assessed using a modified version of the method described in Ref. [36]. Calcium concentration was monitored fluorometrically with Calcium Green 5 N (1 μM; excitation 506 nm, emission 532 nm; Invitrogen). Fluorescence was recorded using a Cary Eclipse spectrofluorometer (Agilent Technologies) with 3 ml quartz cuvettes (1 cm path length). Calcium chloride was added to the incubation medium immediately before measurements at a final concentration of 10 μM.

### 2.3 Statistical analysis

Statistical analysis and graphing were performed using Origin 8.6 software (OriginLab) as was described previously [14]. All data is presented as mean ± SEM.

## 3 Results

### 3.1 BLAST search of PMCA homologs in human proteome

SERCA and PMCA are closely related P-type Ca^2+^-ATPases belonging to the P2A and P2B subfamilies, respectively, and share a conserved catalytic core comprising the actuator, nucleotide-binding, and phosphorylation domains, together with a ten-transmembrane-helix Ca^2+^-transporting region. To verify the sequence (and structure) similarities and find other potential candidates, we performed a protein BLAST search of human PMCA1 (UniProt: P20020) against the human proteome.

We retrieved the top 100 most similar sequences to PMCA. Among them, the first 39 entries corresponded to PMCA isoforms. Beyond these, the most similar non-PMCA protein identified was the sarcoplasmic/endoplasmic reticulum calcium ATPase 3 (SERCA3), with an E-value of 1 × 10^−83^, followed by SERCA1 and SERCA2 with E-values of 6 × 10^−80^ and 2 × 10^−78^, respectively.

### 3.2 Molecular docking

Based on the BLAST results, we next assessed whether polyP could bind SERCA by performing molecular docking simulations. Since human SERCA1 and SERCA3 lacks experimentally determined crystal structures, we employed AlphaFold2 (AF2) to predict their tertiary structure.

**Fig. 1:**
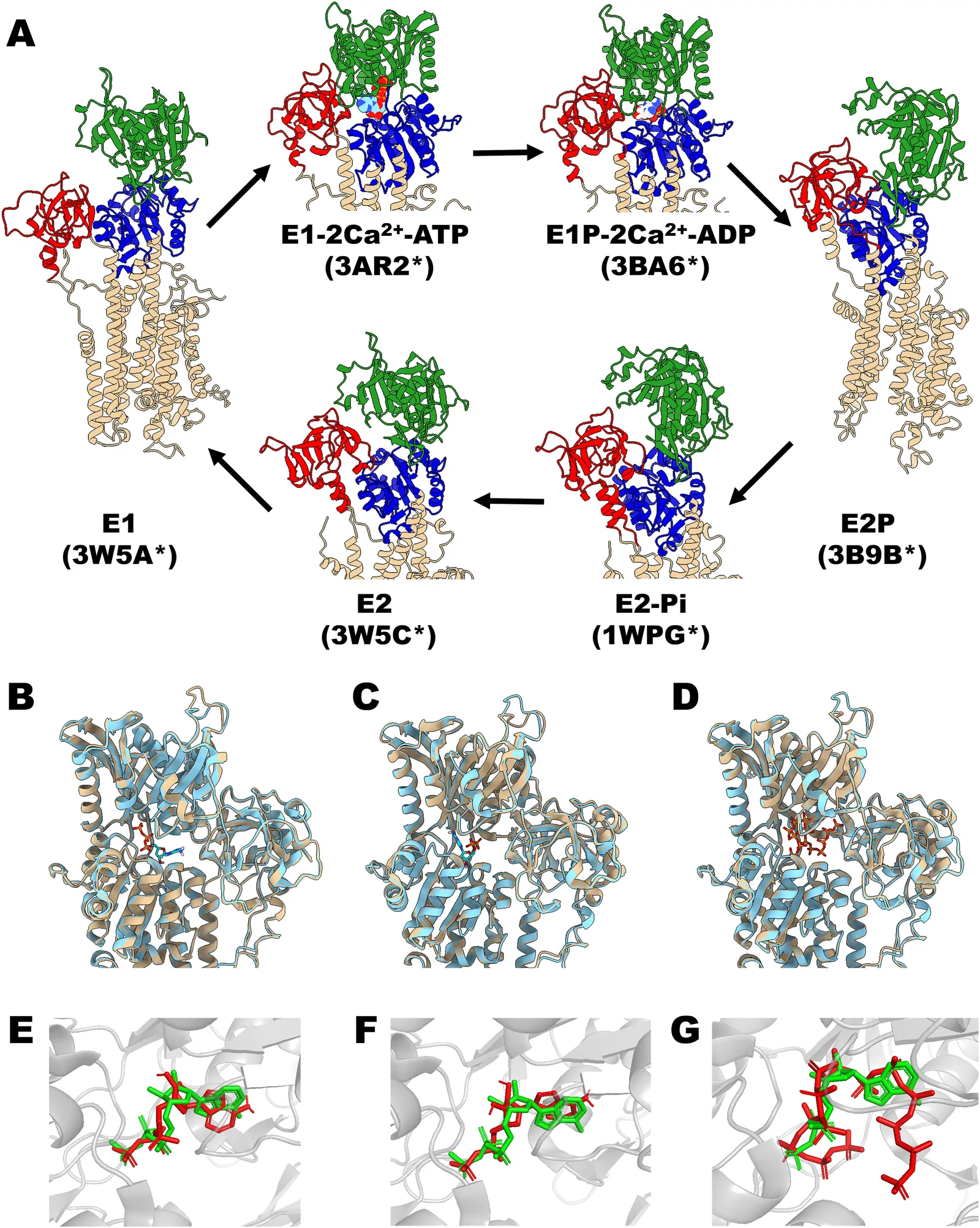
**Predicting polyP-14 binding to SERCA3 in different states. (A)** AlphaFold2 predicted structures for human SERCA3 in 6 well-characterized enzymatic states. **(B,C,D)** docking poses of ATP **(B)**, ADP **(C)**, polyP-14 **(D)** to E1-Ca^2+^-ATP state of human SERCA3; **E, F, G** – close up comparison of the molecular docking result with experimentally determined structure – experimental ligand (AMPPCP – ATP analogue) in green, ligands in red: ATP **(E)** – r.m.s.d. 1.51 Å, ADP **(F)** and polyP-14 **(G).**

Because docking outcomes are strongly influenced by protein conformational states [37,38], we sought to model SERCA1-3 in multiple conformations [39]. To do so, we provided six well-characterized rabbit SERCA structures as masked templates to bias AF2 predictions toward known functional states (Fig. 1A) [13,14]. The resulting AF2-predicted SERCA structures showed low RMSD values (<1 Å) relative to the experimental templates and high confidence scores (pLDDT>90), confirming structural reliability (Suppl. Fig. 1).

We then performed molecular docking of polyP-14 (a polyphosphate chain of 14 units) to the AF2-predicted SERCA models using AutoDock Vina. ATP and ADP were included as control ligands. Docking simulations revealed that polyP-14 binds to SERCA1-3 with negative Vina scores comparable to ATP, the natural substrate of the enzyme. While analyzing the results of docking we found that for SERCA2-3 the strongest binding was observed for E2 and E1–2Ca^2+^–ATP conformations (Vina scores in the range −9.1 and −8.9 kcal/mol), while for SERCA1 the strongest binding was found for the E1–2Ca^2+^–ADP conformation (Vina = −8.8). For ATP, the highest binding affinities were found for the E2, E1, and E1–2Ca^2+^–ATP states of SERCA1-3 (Vina = −8.7) (Table 1).

**Table 1:** Results of molecular docking simulations of ligands to different states and isoforms of SERCA.

Isoform		AutoDock Vina docking scores (kcal/mol)					
Isoform	Ligand	E1	E1-2Ca^2+^-ATP	E1P-2Ca^2+^-ADP	E2P	E2-Pi	E2
**SERCA1**	**ATP**	−8.4	−9.3	−8.7	−7.6	−7.1	−7.4
	**ADP**	−8.1	−9.7	−8.6	−6.7	−7	−7.4
	**polyP-14**	−8.5	−8.3	−8.8	−6.8	−7.2	−8.1
**SERCA2**	**ATP**	−8.2	−9.4	−8.7	−7	−7.3	−7.4
	**ADP**	−8.2	−9	−8.6	−6.8	−7.3	−7.1
	**polyP-14**	−7.4	−9.1	−8.7	−7.2	−7	−7.4
**SERCA3**	**ATP**	−8.7	−8.7	−8	−7.5	−7.7	−8.7
	**ADP**	−8	−8.3	−8.4	−6.8	−7.3	−7.7
	**polyP-14**	−8.3	−8.9	−8.2	−8.4	−7.5	−9

Together, these results indicate that polyP-14 can be accommodated by multiple conformational states of SERCA1–3, with the most favorable docking scores observed for SERCA2 and SERCA3 conformations associated with ATP binding and hydrolysis.

Encouraged by these theoretical predictions, we sought to experimentally validate whether SERCA can functionally utilize polyP as a substrate *in vitro*. To test this hypothesis, we assessed the calcium transport activity of isolated SERCA-containing microsomal vesicles.

## 4 Assessment of polyP utilization by SERCA-containing vesicles for calcium transport

**Fig. 2:**
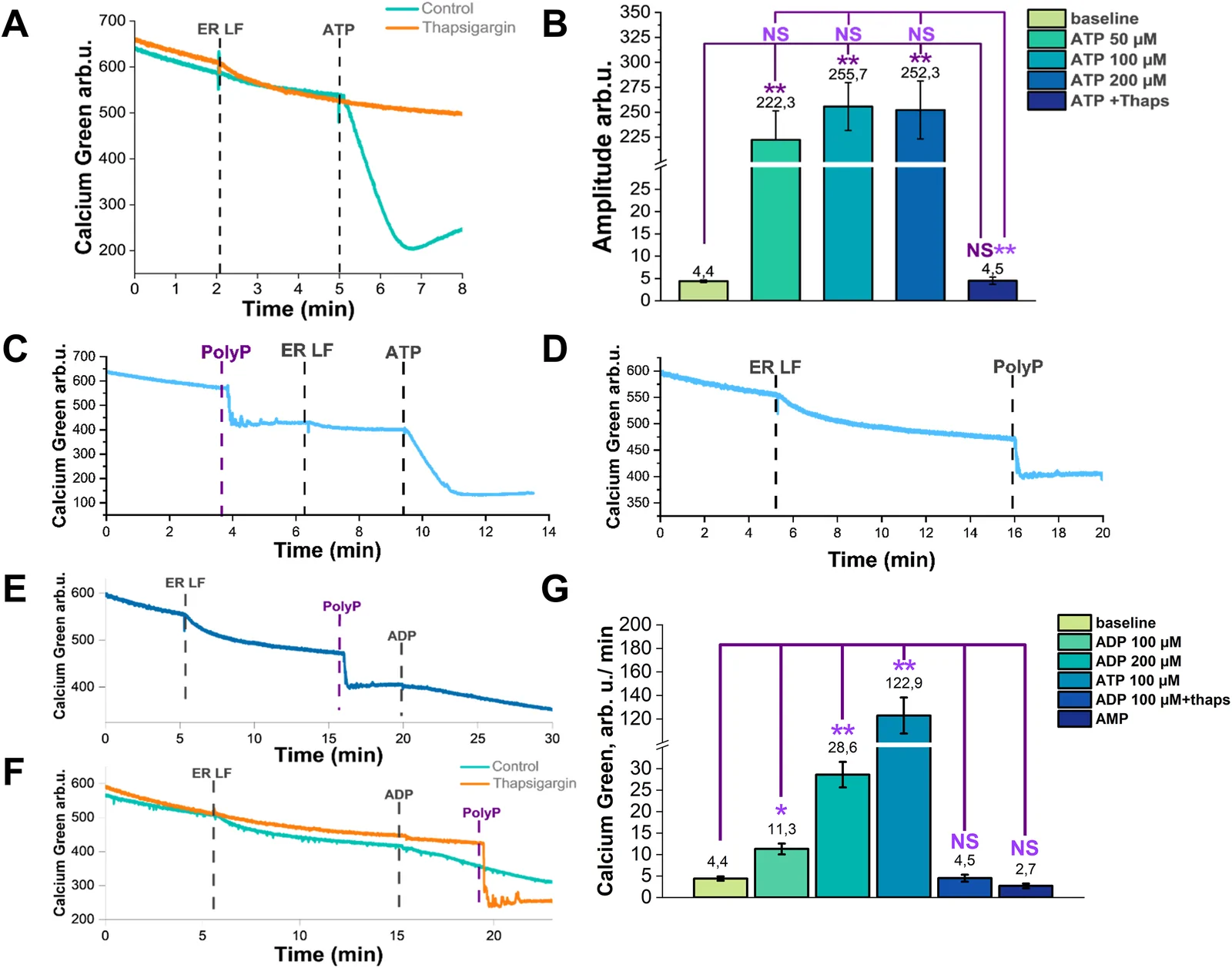
**Functional characterization of SERCA-containing vesicles in the presence of polyP. (A)** Representative traces of Calcium Green 5 N fluorescence showing Ca^2+^ uptake into vesicles. **(B)** Quantification of total Ca^2+^ uptake at varying ATP concentrations, demonstrating no significant differences, consistent with saturation of vesicles under the experimental conditions. **(C)** Representative trace showing Ca^2+^ dynamics following addition of polyphosphate (polyP) and subsequent ADP, resulting in further decrease in Ca^2+^ concentration. **(D)** Control experiment of ADP dependent decrease in Ca^2+^ concentration, which is fully inhibited by thapsigargin. **(E)** Quantification of SERCA activity in the presence of different nucleotide conditions. Number of experiments - ATP 50 μM, n = 7; ATP 100 μM, n = 18; ATP 200 μM, n = 5; ATP 100 μM + thapsigargin, n = 4 ADP 100 μM, n = 7; ADP 200 μM, n = 4; ATP 100 μM, n = 18; ATP 100 μM + thapsigargin, n = 4; AMP 100 μM, n = 4. Statistical significance is indicated as follows: *p < 0.05, **p < 0.01, NS – not significant.

To assess the functional status of isolated SERCA-containing vesicles at the beginning of each experiment, fluorescence intensity of Calcium Green-5N was monitored for 2 min in the presence of 10 μM Ca^2+^ to ensure signal stability and exclude spontaneous fluctuations. Addition of the vesicles did not significantly affect the fluorescence intensity of Calcium Green-5N (Fig. 2A). Following addition of ATP, resulted in a sharp decrease in fluorescence intensity, indicating a reduction in free calcium concentration in the cuvette (Fig. 2A).

Application of the selective SERCA inhibitor thapsigargin completely abolished the ATP-dependent decrease in Ca^2+^ concentration (Fig. 2A). These results demonstrate that the isolated vesicles actively uptake Ca^2+^ in an ATP-dependent manner, and that this process is mediated specifically by SERCA activity. The SERCA activity was independent on the ATP concentration applied (Fig. 2B), suggesting that the tested ATP concentrations were sufficient to achieve maximal calcium loading of the vesicles.

Reusch et al. previously demonstrated that PMCA can function as a polyphosphate kinase [26]. We therefore sought to determine whether SERCA possesses similar activity. Application of 10 μM polyP in the presence of vesicles caused a rapid decrease in free Ca^2+^ concentration in the cuvette (Fig. 2C). Further ATP addition induced a further decrease in Ca^2+^ levels, similar to its effect in the absence of polyP. To exclude any artifacts, in the next experiment polyP was added without vesicles and produced a comparable decrease in free Ca^2+^ concentration (Fig. 2D). Since polyP is a polyanion capable of binding divalent cations, including Ca^2+^, these data indicate that the observed effect reflects Ca^2+^ chelation rather than SERCA-mediated transport. However, if SERCA exhibits polyphosphate kinase activity, then in a system containing SERCA, polyP, and adenosine diphosphate (ADP), ATP synthesis may occur. The ATP generated could subsequently be used by SERCA to transport calcium into the vesicle lumen, resulting in a decrease in Calcium Green-5N fluorescence intensity. The addition of polyP to the cuvette also reduced calcium concentration. Notably, subsequent addition of 100 μM ADP led to a further decrease in calcium concentration (Fig. 2E). Although these observations were consistent with our hypothesis, control experiments were performed to exclude potential artifacts. In the absence of polyP, the addition of ADP alone also resulted in a comparable decrease in calcium concentration (Fig. 2F). Importantly, ADP did not affect calcium concentration in the absence of SERCA, indicating that ADP itself does not chelate calcium. Application of thapsigargin completely abolished the ADP-dependent calcium transport into vesicles (Fig. 2F), confirming that the observed process is mediated by SERCA activity. Further experiments demonstrated that SERCA activity depended on ADP concentration: increasing ADP from 100 μM to 200 μM resulted in a 2.5-fold increase in the rate of calcium uptake (Fig. 2E). In contrast, the addition of adenosine monophosphate (AMP) at 100 μM did not affect calcium concentration (Fig. 2E). Interestingly, Ca^2+^ transport was observed only in the presence of ADP potassium salt, but not ADP sodium salt, although both compounds were obtained from Sigma.

In this series of experiments, SERCA did not support Ca^2+^ transport when polyP was provided as the sole phosphoryl donor, indicating that polyP cannot substitute for ATP as an energy source for SERCA activity. In addition, no evidence was obtained for SERCA-mediated polyphosphate kinase activity or ATP synthesis, in contrast to previous observations for PMCA. Interestingly, ADP supported Ca^2+^ transport into the ER lumen under our experimental conditions; however, this observation requires further confirmation.

## 5 Evaluation of Ca^2+^ binding by polyP – *in vitro* and *in silico* studies

Given that polyP is polyanionic molecules capable of binding various cations, including Ca^2+^, in the next series of experiments we tested its ability to bind Ca^2+^. To test this, we performed experiments with varying concentrations of polyP in the absence of SERCA-containing vesicles.

**Fig. 3:**
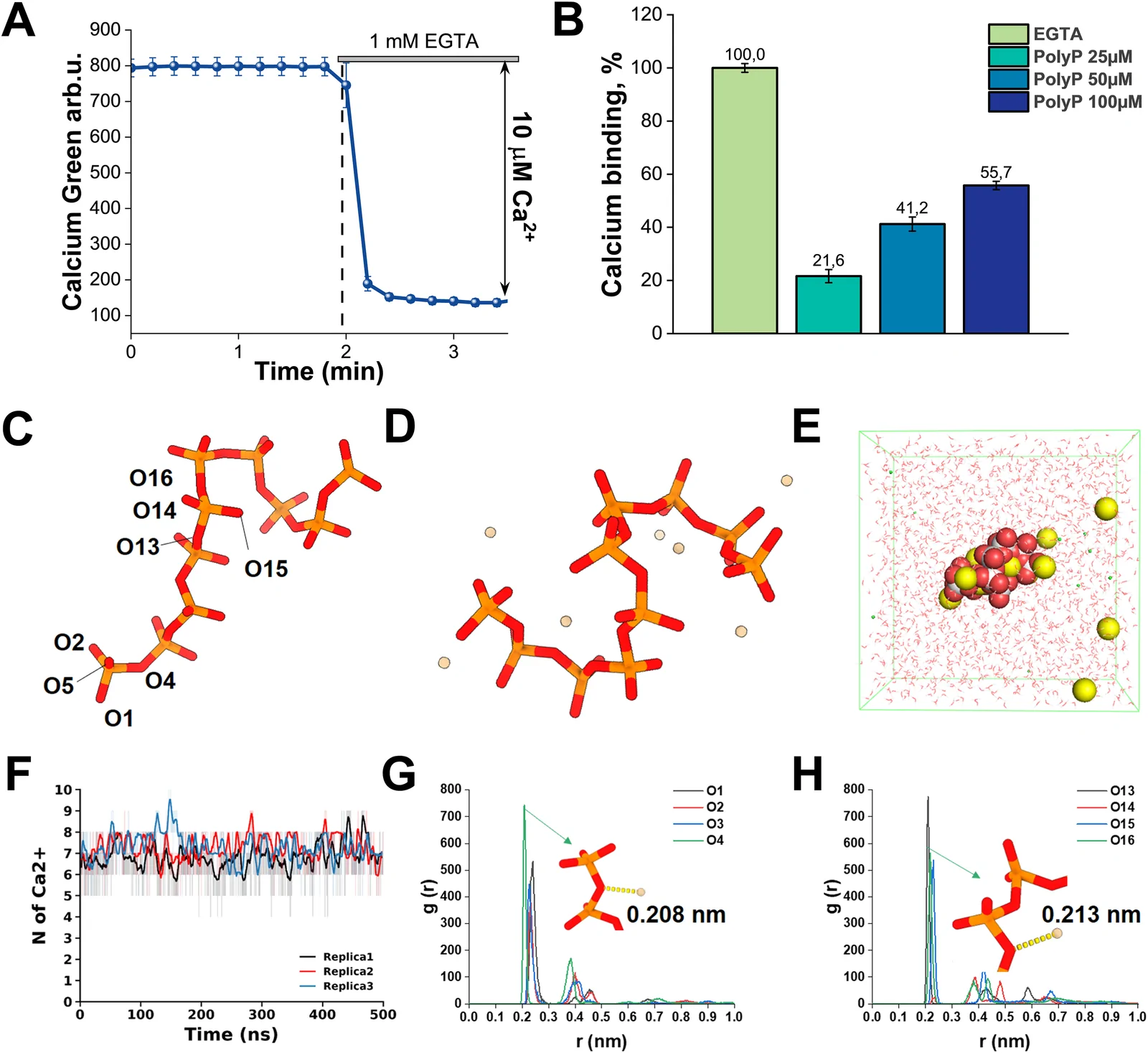
**Polyphosphate-induced Ca^2+^ chelation is independent of SERCA activity. (A)** Calibration of maximal Ca^2+^ binding using 1 mM EGTA in the absence of vesicles, defining 100% Ca^2+^ chelation. **(B)** Quantification of Ca^2+^ binding at increasing polyP concentrations (25–100 μM) in the absence of vesicles. Number of experiments - EGTA, n = 6; polyP 25 μM, n = 5; polyP 50 μM, n = 7; polyP 100 μM, n = 10. **(C,D)** The structure of PolyP-14 at 0 ns (**C**) and 500 ns (**D**) of simulation with marked oxygen atoms used in rdf analysis. **(E)** the final snapshot of simulation box, depicting ions, PolyP and water molecules; **(F)** the evolution of Ca^2+^ ions near the PolyP molecule during the course of simulations in 3 replicates; **(G,H)** radial distribution function plots for Ca^2+^ ions coordination by oxygen atoms of the distal phosphate group (**E**) and middle phosphate group (**F**).

To establish a calibration reference for our measurements, total calcium was chelated by adding 1 mM EGTA to cuvette containing 10 μM Ca^2+^ and Calcium Green-5N, in the absence of SERCA-containing vesicles. This condition was defined as 100% calcium binding (Fig. 3A). Application of different polyP concentration into the cuvette in the absence of SERCA-containing vesicles showed concentration dependent decrease of Calcium Green-5N, which we interpreted as decrease in free calcium concentration (Fig. 3B).

To further study the ability of polyP to directly bind Ca^2+^ ions, we performed molecular dynamics (MD) simulations using the GROMACS software. The MD simulations were carried out for 500 ns in 3 independent replicates with different initial ion distributions, after which we calculated the number of Ca^2+^ ions bound to polyP-14. We found that polyP binds Ca^2+^ ions instantly at the beginning of all MD simulations, and 7–9 Ca^2+^ ions remain stably associated with the polyP molecule throughout the simulations (Fig. 3B–D). Analyzing the coordination of Ca^2+^ ions to polyP, we identified two distinct mechanisms of coordination: binding by the outer oxygen atoms and coordination by four oxygen atoms forming phosphoanhydride bonds (Fig. 1A). To examine the differences in calcium coordination by various oxygen atoms in polyP, we performed a radial distribution function (rdf) analysis. We found that inter-P oxygen atoms coordinate Ca^2+^ ions at shorter distances (0.208 nm), while side oxygen atoms bind Ca^2+^ ions at slightly larger distances (0.226–0.24 nm). We also observed that polyP-14 can bind Ca^2+^ ions indirectly via water molecules at longer distances of 0.34–0.4 nm (Fig. 3E and F).

Thus, our experiments demonstrated that polyP can efficiently bind Ca^2+^ in solution, as shown by both *in vitro* and *in silico* approaches.

## 6 Discussion

In this study, we examined whether inorganic polyphosphate can support SERCA-dependent Ca^2+^ transport or act as a phosphoryl donor in a polyphosphate kinase-like reaction, as previously reported for PMCA. Using SR vesicles isolated from rabbit skeletal muscle, we show that polyP does not substitute for ATP as an energy source for SERCA and does not support detectable SERCA-associated polyphosphate kinase-like activity. Instead, the polyP-induced decrease in free Ca^2+^ by direct Ca^2+^ chelation rather than Ca^2+^ transport.

This conclusion is important because molecular docking predicted favorable binding of polyP-14 to SERCA1–3, with Vina scores comparable to ATP and ADP. However, because docking scores are influenced by ligand size, charge, and chemical composition, the Vina scores obtained for polyP-14 cannot be directly compared quantitatively with those obtained for ATP or ADP. Therefore, we interpreted our docking results as indicating that polyP-14 can adopt favorable binding poses within the SERCA models, rather than as evidence of binding affinities comparable to those of adenine nucleotides. Thus, our data highlight the distinction between predicted ligand binding and functional catalytic utilization. Although polyP may be accommodated within the nucleotide-binding region, its extended phosphate chain and high negative charge density may prevent the geometry required for productive phosphoryl transfer to the conserved aspartate residue of the P-domain.

The functional experiments used vesicles derived from rabbit skeletal muscle, expected to be enriched mainly in SERCA1a [40]. Therefore, the present conclusions primarily apply to SERCA1a under the tested conditions. Because polyphosphate kinase-like activity of PMCA was originally reported in human erythrocytes, and because SERCA3 is expressed in blood-derived, endothelial, and secretory cell types, future studies should determine whether SERCA3 responds differently to polyP in its native cellular environment. This cellular context may be particularly relevant because polyP is abundant in blood-related and endothelial cells, where it contributes to coagulation and inflammatory signaling, and platelet polyP concentrations may reach up to 130 mM [41].

A key, but not unexpected, methodological finding is that polyP chelates Ca^2+^ in solution. *In vitro*, polyP reduced Calcium Green-5N fluorescence in a dose-dependent manner even in the absence of vesicles, whereas 500 ns molecular dynamics simulations showed stable coordination of 7–9 Ca^2+^ ions per polyP-14 chain. Therefore, changes in free Ca^2+^ must be carefully controlled when interpreting polyP effects in Ca^2+^-dependent biological systems, which was also described recently by Muller and colleagues [42].

Unexpectedly, ADP, but not AMP, induced concentration-dependent, thapsigargin-sensitive Ca^2+^ uptake. Although thapsigargin sensitivity indicates the involvement of SERCA, the present experiments do not establish whether ADP contributes to Ca^2+^ transport directly or through an indirect mechanism. Possible contributions from K^+^, ATP contamination of the ADP preparation, or ATP-generating enzymatic activities associated with the microsomal fraction cannot be excluded and require further investigation.

Thus, our findings demonstrate that polyP is a potent Ca^2+^ chelator but cannot substitute for ATP as an energy source for SERCA.

## CRediT authorship contribution statement

**F.A. Khasanov:** Investigation, Formal analysis. **F.R. Rustamova:** Visualization, Investigation, Formal analysis. **E.P. Semernya:** Investigation. **L.M. Akhmadkhojaeva:** Investigation, Formal analysis. **K.S. Nebesnaya:** Project administration, Data curation. **K.R. Rustamov:** Writing – review & editing, Writing – original draft, Visualization, Investigation, Formal analysis. **A.Y. Baev:** Writing – review & editing, Writing – original draft, Supervision, Investigation, Funding acquisition, Formal analysis, Conceptualization.

## Declaration of competing interests

The authors declare that they have no known competing financial interests or personal relationships that could have appeared to influence the work reported in this paper.

## Data Availability

Data will be made available on request.
