# Supplementary material for: Inorganic polyphosphate chelates Ca2+ but cannot substitute for ATP as an energy source for SERCA
Source: Biochem Biophys Rep. 2026 Sep 14;48:102796. doi: 10.1016/j.bbrep.2026.102796 (PMC13594633; doi:10.1016/j.bbrep.2026.102796)
Supplement: Supplementary file 1 [file mmc1.docx]

**Supplementary Figure 1. AlphaFold2 predicted structures of human SERCA in 6 different states using rabbit SERCA as masked template.**

**Supplementary Table. AutoDock Vina docking grid-box parameters for all SERCA targets**

| **Model** | **Isoform** | **Conformational state** | **Grid center X (Å)** | **Grid center Y (Å)** | **Grid center Z (Å)** | **Grid size (Å)** | **Exhaustiveness** |
| --- | --- | --- | --- | --- | --- | --- | --- |
| AlphaFold2 SERCA1 | SERCA1 | E1 | -23.313 | -5.082 | 15.787 | 15 × 15 × 15 | 8 |
| AlphaFold2 SERCA1 | SERCA1 | E1·2Ca²⁺·ATP | -21.532 | -4.589 | 11.719 | 15 × 15 × 15 | 8 |
| AlphaFold2 SERCA1 | SERCA1 | E1P·2Ca²⁺·ADP | -19.606 | -6.350 | 11.956 | 15 × 15 × 15 | 8 |
| AlphaFold2 SERCA1 | SERCA1 | E2P | -24.931 | -5.756 | 21.563 | 15 × 15 × 15 | 8 |
| AlphaFold2 SERCA1 | SERCA1 | E2·Pi | -22.438 | -3.499 | 21.931 | 15 × 15 × 15 | 8 |
| AlphaFold2 SERCA1 | SERCA1 | E2 | -21.383 | -4.687 | 16.857 | 15 × 15 × 15 | 8 |
| AlphaFold2 SERCA2 | SERCA2 | E1 | -21.589 | -8.547 | 19.125 | 15 × 15 × 15 | 8 |
| AlphaFold2 SERCA2 | SERCA2 | E1·2Ca²⁺·ATP | -19.705 | -9.127 | 15.656 | 15 × 15 × 15 | 8 |
| AlphaFold2 SERCA2 | SERCA2 | E1P·2Ca²⁺·ADP | -18.354 | -8.971 | 15.277 | 15 × 15 × 15 | 8 |
| AlphaFold2 SERCA2 | SERCA2 | E2P | -23.807 | -5.920 | 24.076 | 15 × 15 × 15 | 8 |
| AlphaFold2 SERCA2 | SERCA2 | E2·Pi | -22.638 | -1.447 | 24.170 | 15 × 15 × 15 | 8 |
| AlphaFold2 SERCA2 | SERCA2 | E2 | -20.619 | -5.387 | 19.758 | 15 × 15 × 15 | 8 |
| AlphaFold2 SERCA3 | SERCA3 | E1·2Ca²⁺·ATP | -9.475 | -7.233 | 26.058 | 15 × 15 × 15 | 8 |
| AlphaFold2 SERCA3 | SERCA3 | E1P·2Ca²⁺·ADP | -8.838 | -5.704 | 25.395 | 15 × 15 × 15 | 8 |
| AlphaFold2 SERCA3 | SERCA3 | E2P | -17.226 | -7.575 | 31.121 | 15 × 15 × 15 | 8 |
| AlphaFold2 SERCA3 | SERCA3 | E2·Pi | -16.380 | -1.465 | 29.894 | 15 × 15 × 15 | 8 |
| AlphaFold2 SERCA3 | SERCA3 | E2 | -18.165 | -8.603 | 23.734 | 15 × 15 × 15 | 8 |
| 3AR2 (experimental) | SERCA1a (rabbit) | E1-2Ca-ATP (native, AMPPCP-bound) | 73.364 | 28.661 | 61.769 | 15 × 15 × 15 | 8 |

***Molecular docking of ligands to SERCA.***

To investigate **polyP binding to SERCA**, molecular docking simulations were performed using **AutoDock Vina**. Docking was carried out for **ATP, ADP,** and **polyP-14** ligands against **six AlphaFold2-predicted conformations** of SERCA representing distinct functional states. Ligand geometries were constructed and optimized from SMILES representation using RDKit Python package and Open Babel software; AF2 predicted protein structures were prepared by assigning polar hydrogen atoms as well as partial charges using standard **Vina prepare_receptor command**. The docking grid was centered on the **ATP-binding pocket** of SERCA with dimensions of **15 × 15 × 15 Å**. Each simulation was performed using a **random seed** and an **exhaustiveness parameter of 8**.

***Molecular dynamics simulations.***

To explore the interactions between polyP-14 and Ca^2^+ ions, we performed molecular dynamics (MD) simulations using GROMACS. System preparation was carried out via the CHARMM-GUI web server. Parameters and topology files for polyP-14 were generated using CGenFF. The polyP-14 molecule was then solvated in a TIP3P water box, and 20 Ca^2^+ and 10 Cl- counterions were added to neutralize the system. Energy minimization and equilibration were performed following the default CHARMM-GUI protocol: systems were minimized for 5000 step using the steepest descent algorithm, and the following NVT equilibration was performed. Subsequently, a 500 ns simulation was performed in NPT ensemble for all our systems using Partial Mesh Ewald (PME) methods to monitor long-range electrostatics. For temperature coupling at 310.1 K Nose-Hoover thermostat was used. And the pressure was preserved with the help of Parrinello-Rahman barostat. All covalent bonds were constrained to the equilibrium length by the LINCS algorithm. The number of calcium ions coordinated to polyP-14 (within the distance of 2.5 Å) was calculated using MDAnalysis, and the radial distribution function (RDF) of Ca^2^+ ions relative to selected oxygen atoms of polyP-14 was computed using built-in GROMACS tools.
